
JOURNAL INFORMATION
==============================
NLM Title Abbreviation: J Can Health Libr Assoc
Journal ID: J Can Health Libr Assoc
NLM Journal ID: 101229936
Journal ID: JCHLA

The Journal of the Canadian Health Libraries Association

EISSN: 1708-6892
Publisher: Canadian Health Libraries Association / Association des bibliotèques de la santé du Canada

ARTICLE INFORMATION
==============================
PMCID: PMC11485159
DOI: 10.29173/jchla29790
Article ID: JCHLA-45-126
Article version: 1
Subjects: Chla 2024 Conference Interactive Workshops / Absc Congrès 2024 Ateliers Interactifs

CHLA 2024 CONFERENCE INTERACTIVE WORKSHOPS / ABSC CONGRÈS 2024 ATELIERS INTERACTIFS

Electronic publication date: 2024 Aug 1
Publication date: 2024 Aug
Volume: 45
Issue: 2
First page: 126
Last page: 126
License: This article is distributed under a Creative Commons Attribution License: https://creativecommons.org/licenses/by/4.0/
License URL: https://creativecommons.org/licenses/by/4.0/

==============================
JOURNAL INFORMATION
==============================
NLM Title Abbreviation: J Can Health Libr Assoc
Journal ID: J Can Health Libr Assoc
NLM Journal ID: 101229936
Journal ID: JCHLA

The Journal of the Canadian Health Libraries Association

EISSN: 1708-6892
Publisher: Canadian Health Libraries Association / Association des bibliotèques de la santé du Canada

ARTICLE INFORMATION
==============================
Subjects: IW = Interactive Workshops

IW1. Dedicated time for reading, brainstorming, writing, or reworking a project!

Hoogland Margaret 1
Ferri Anna 2
Neilson Christine 3
Atwood Gary 4
1 University of Toledo;
2 Colorado State University;
3 University of Manitoba;
4 University of Vermont

JOURNAL INFORMATION
==============================
NLM Title Abbreviation: J Can Health Libr Assoc
Journal ID: J Can Health Libr Assoc
NLM Journal ID: 101229936
Journal ID: JCHLA

The Journal of the Canadian Health Libraries Association

EISSN: 1708-6892
Publisher: Canadian Health Libraries Association / Association des bibliotèques de la santé du Canada

ARTICLE INFORMATION
==============================
Subjects: IW = Interactive Workshops

IW2. Organizing for power in your health sciences library: a workshop to utilize labour organizing techniques to engage with health library patrons, and advocate for library workers

Dingwall Orvie
University of Manitoba

